# The buildup of an urge in obsessive–compulsive disorder: Behavioral and neuroimaging correlates

**DOI:** 10.1002/hbm.24898

**Published:** 2020-01-09

**Authors:** Emily R. Stern, Carina Brown, Molly Ludlow, Rebbia Shahab, Katherine Collins, Alexis Lieval, Russell H. Tobe, Dan V. Iosifescu, Katherine E. Burdick, Lazar Fleysher

**Affiliations:** ^1^ Department of Psychiatry New York University School of Medicine New York New York; ^2^ Nathan Kline Institute for Psychiatric Research Orangeburg New York; ^3^ Department of Psychiatry Icahn School of Medicine at Mount Sinai New York New York; ^4^ Department of Psychiatry Brigham and Women's Hospital Boston Massachusetts; ^5^ Department of Radiology Icahn School of Medicine at Mount Sinai New York New York

**Keywords:** fMRI, insula, interoception, obsessive–compulsive disorder, repetitive behavior, sensorimotor, suppress, urge

## Abstract

Obsessive–compulsive disorder (OCD) is highly heterogeneous. While obsessions often involve fear of harm, many patients report uncomfortable sensations and/or urges that drive repetitive behaviors in the absence of a specific fear. Prior work suggests that urges in OCD may be similar to everyday “urges‐for‐action” (UFA) such as the urge to blink, swallow, or scratch, but very little work has investigated the pathophysiology underlying urges in OCD. In the current study, we used an urge‐to‐blink approach to model sensory‐based urges that could be experimentally elicited and compared across patients and controls using the same task stimuli. OCD patients and controls suppressed eye blinking over a period of 60 s, alternating with free blinking blocks, while brain activity was measured using functional magnetic resonance imaging. OCD patients showed significantly increased activation in several regions during the early phase of eyeblink suppression (first 30 s), including mid‐cingulate, insula, striatum, parietal cortex, and occipital cortex, with lingering group differences in parietal and occipital regions during late eyeblink suppression (last 30 s). There were no differences in brain activation during free blinking blocks, and no conditions where OCD patients showed reduced activation compared to controls. In an exploratory analysis of blink counts performed in a subset of subjects, OCD patients were less successful than controls in suppressing blinks. These data indicate that OCD patients exhibit altered brain function and behavior when experiencing and suppressing the urge to blink, raising the possibility that the disorder is associated with a general abnormality in the UFA system that could ultimately be targeted by future treatments.

## 1 INTRODUCTION

Obsessive–compulsive disorder (OCD) is diagnosed by the presence of obsessions (intrusive thoughts, impulses, or images that cause anxiety) and/or compulsions (repetitive behaviors typically performed in response to obsessions). OCD is highly heterogeneous, with many patients reporting uncomfortable sensations and/or urges that precede and drive their repetitive behaviors in the absence of any specific fear or thought (Brandt et al., 2018;Ferrao et al., 2012; Lee et al., 2009; Rosario et al., 2009; Shavitt et al., 2014). Parallels have been drawn between these sensory‐based urges prior to compulsive behavior in OCD and premonitory urges prior to tics in Tourette's disorder (TD) (Miguel et al., 2000; Rosario et al., 2009), both of which fall within the category of “sensory phenomena” (SP) that are reported in 60–70% of OCD patients (Ferrao et al., 2012; Lee et al., 2009; Rosario et al., 2009; Shavitt et al., 2014) and >90% of TD patients (Cavanna, Black, Hallett, & Voon, 2017; Leckman, Walker, & Cohen, 1993; Reese et al., 2014).

Prior work has suggested there may be phenomenological similarities between the urges experienced in OCD and TD and everyday “urges‐for‐action” (UFA; Berman, Horovitz, Morel, & Hallett, 2012; Jackson, Parkinson, Kim, Schuermann, & Eickhoff, 2011), which are sensations that drive an individual to perform a behavior, such as the urge to blink, swallow, or scratch. Functional magnetic resonance imaging (fMRI) studies examining UFA in healthy individuals have linked them to activation of the insula and sensorimotor cortical regions (mid‐cingulate cortex, supplementary motor area [SMA], precentral and postcentral gyri) (Berman et al., 2012; Holle, Warne, Seth, Critchley, & Ward, 2012; Jackson et al., 2011; Lerner et al., 2009; Mazzone, Cole, Ando, Egan, & Farrell, 2011), areas frequently linked to interoception, somatosensation, and movement preparation (Critchley, Wiens, Rotshtein, Ohman, & Dolan, 2004; Lee, Chang, & Roh, 1999; Schulz, 2016). These same regions (insula, mid‐cingulate, SMA, precentral gyrus, and postcentral gyrus) are activated right before tic onset in TD patients (Bohlhalter et al., 2006; Neuner et al., 2014). Greater connectivity between insula and SMA at rest (Tinaz et al., 2014) and altered gray matter thickness in insula and sensorimotor cortex (Draganski et al., 2010; Draper, Jackson, Morgan, & Jackson, 2016) have been associated with increased premonitory urges in TD. Using eyeblink suppression as an experimental model for a sensory‐based urge that can be reliably elicited in both patients and controls, Mazzone et al. (2010) found that greater tic severity in TD was associated with reduced activity during eyeblink suppression in inferior frontal gyrus, superior temporal gyrus, and putamen, effects that were interpreted as reflecting impaired inhibitory control. Although not a direct examination of urges, investigations into the neural correlates of SP in OCD have linked these symptoms to gray matter volume in sensorimotor cortex (precentral/postcentral gyri) (Subira et al., 2015) and functional activation of the mid‐insula and somatosensory cortex (Brown et al., 2019).

Despite the multiple studies looking at urges in healthy controls and patients with tics, few studies have investigated urges in OCD, and none have examined brain functioning in relation to the buildup of an UFA in the disorder. In the present study, we address this gap by examining brain function in OCD patients and controls during an fMRI task where periods of eyeblink suppression alternate with free blinking blocks, similar to previous work in healthy individuals and TD patients (Berman et al., 2012; Mazzone et al., 2010). This relatively simple paradigm experimentally elicits the urge to blink (Berman et al., 2012; Brandt et al., 2016; Brandt et al., 2018), allowing for the comparison of urge‐related activity between OCD patients and controls using the same task stimuli. In a subset of participants, we investigated the number of eyeblinks made during blink suppression and free blinking periods to determine whether the groups differed in suppression success. We hypothesized that OCD patients would show greater activity in the UFA network including insular and sensorimotor regions (precentral and postcentral gyri, cingulate cortex, SMA) compared to controls during blink suppression, and that this effect would be stronger for patients with prominent SP. Findings from this investigation will help elucidate the neural mechanisms associated with an important yet understudied feature of OCD, which could ultimately contribute to the development of future treatments aimed at targeting pathological urges through the modulation of their underlying circuitry.

## 2 METHODS

### 2.1 Subjects and procedure

Patients were recruited at three locations and scanned at two of these locations between April 2017 and 2019. During that time frame, 50 patients with OCD completed the study, 19 of which were recruited and scanned at the Icahn School of Medicine at Mount Sinai (ISMMS), 13 of which were recruited and scanned at the Nathan Kline Institute for Psychiatric Research (NKI), and 18 of which were recruited at the New York University School of Medicine (NYUSoM) and also scanned at NKI. Twenty‐four healthy controls also completed the study (11 recruited and scanned at ISMMS, six recruited and scanned at NKI, and seven recruited at NYUSoM and scanned at NKI). Data from four OCD patients and one control participant were excluded (three patients recruited and scanned at ISMMS were excluded due to technical error associated with the scanner sequence; one patient at ISMMS fell asleep during the scan; and one control recruited and scanned at NKI fell asleep during the scan). Final data were analyzed from 46 OCD patients and 23 controls. All patients met DSM‐5 criteria for OCD and were excluded for lifetime presence of bipolar disorder or schizophrenia spectrum disorder. Out of the 46 patients, 31 (67%) had at least one current comorbid Axis I disorder including generalized anxiety disorder (*n* = 17), panic disorder (*n* = 7), attention deficit hyperactivity disorder (*n* = 6), and social anxiety disorder (*n* = 6). Less frequent current comorbidities included excoriation disorder (*n* = 4), agoraphobia (*n* = 4), trichotillomania (*n* = 3), body dysmorphic disorder (*n* = 3), alcohol use disorder mild (*n* = 3), major depressive disorder (*n* = 2), illness anxiety disorder (*n* = 2), hoarding disorder (*n* = 2), TD (*n* = 2), persistent tic disorder (motor) (*n* = 1), suicide behavior disorder (*n* = 1), and binge eating disorder mild (*n* = 1). Out of the 46 patients, 22 (47.8%) were not taking psychotropic medications; the remaining 24 patients were taking serotonin reuptake inhibitors (*n* = 22), risperidone (*n* = 1), trazodone (*n* = 2), lisdexamfetamine dimesylate (*n* = 1), dextroamphetamine–amphetamine (*n* = 1), clonidine (*n* = 1), nortriptyline (*n* = 1), lamotrigine (*n* = 1), and benzodiazepines as needed (*n* = 4, which patients refrained from taking on the day of scanning). Diagnoses were made by a trained rater using the Mini International Neuropsychiatric Interview (Sheehan et al., 1998). Overall severity of obsessive–compulsive symptoms was measured using the total score from the Yale‐Brown Obsessive–Compulsive Scale (Y‐BOCS, (Goodman et al., 1989). SP were assessed using the SP Scale (SPS, (Rosario et al., 2009; Sampaio, McCarthy, Mancuso, Stewart, & Geller, 2014). The SPS is a semistructured interview containing a checklist composed of examples of different types of SP preceding or occurring at the same time as repetitive behaviors and encompasses all previous descriptions in the literature, including physical sensations, “not just right” sensations, incompleteness, general energy or inner tension buildup, and urges. After individuals endorse specific checklist items, severity is measured through ratings of frequency, distress, and interference on 6‐point scales (0–5). Possible total scores range from 0 (no SP) to 15 (severe SP). The SPS shows excellent convergent validity with an open clinical interview (the gold standard), very good discriminative validity, and high inter‐rater reliability (Rosario et al., 2009), and has been used to measure severity of SP in several OCD patient samples (Brown et al., 2019; Ferrao et al., 2012; Lee et al., 2009; Miguel et al., 2000; Rosario et al., 2009; Subira et al., 2015). Self‐reported severity of general depressive and anxiety symptoms were assessed with the Quick Inventory of Depression Symptomatology (QIDS; Rush et al., 2003) and the Beck Anxiety Inventory (BAI; Beck, Epstein, Brown, & Steer, 1988), respectively.

### 2.2 UFA task

This task elicits an UFA by asking participants to suppress eye blinking alternating with free blinking. The rationale for selecting this task is based on published work using eyeblink suppression as a model for sensory‐based urges in OCD and TD (Berman et al., 2012; Botteron et al., 2019; Cavanna et al., 2017; Jackson et al., 2011; Lerner et al., 2009; Mazzone et al., 2010). The design we used is similar to that employed by Berman et al. (Berman et al., 2012), with blocks of normal or free blinking (30 s) alternating with longer blink suppression periods (60 s). During free blinking blinks, subjects see the instruction “NORMAL” on the screen, which they have been told means they should blink as they normally would. During blink suppression, subjects see the instruction “HOLD” on the screen, which they have been instructed means they should try to withhold or suppress blinking for as long as the instruction is displayed. After the 60 s, there is a recovery period where subjects see the phrase “OK TO BLINK” on the screen. This instruction means they are now permitted to blink as much as they wish (4 s), following which they rate the strength of their urge to blink during the prior “HOLD” period on a 5‐point scale (1=“none at all”, 5 = “extreme”) (4 s). After each rating there is a jittered intertrial interval consisting of a fixation cross from 2 to 5 s (plus any leftover time from the rating screen if the rating is made before the full 4 s have elapsed). Eight blocks each of blink suppression and free blinking are presented over two runs. Results from a similar task in healthy controls indicated that all subjects experienced an urge to blink over the 60 s suppression period, and the majority was able to withhold blinking during that time (Lerner et al., 2009). Following the approach employed by Berman et al. (2012), subjects were instructed to return immediately to blink suppression should any accidental blinks occur during the “HOLD” period. An eye‐tracking device (Eyelink 1000 Plus, SR Research, ON, Canada) was used to measure eyeblinks during the task based on a system that tags eyeblinks as instances when the pupil is missing, very small, or distorted by eyelid occlusion.

### 2.3 Neuroimaging data acquisition and preprocessing

All MRI scanning occurred on Siemens 3T scanners (MAGNETOM Skyra at ISMMS and MAGNETOM TrioTim at NKI) with sequences harmonized between the two scanning sites. Functional blood oxygen level dependent (BOLD) data were acquired using a 32‐channel head coil with a high‐resolution multiband‐accelerated echo‐planar sequence for full brain coverage (Repetition time [TR] = 1,000 ms, flip angle = 60°, field‐of‐view = 228 mm, 72 slices, 2.1‐mm thickness, acceleration factor = 6). In order to match all other aspects of the sequences as closely as possible, the echo times (TEs) were slightly different between the two scanning sites (TE = 25 ms at ISMMS and TE = 25.4 ms at NKI). Task runs were acquired in an anterior–posterior phase encoding direction; two phase‐encode‐reversed fieldmap pairs were acquired to use for distortion correction (“topup” in FSL). Preprocessing was performed using a combination of Statistical Parametric Mapping v.12, scripts taken from the Human Connectome Project preprocessing pipeline (Glasser et al., 2013), and FSL v. 5.0.10, and included gradient nonlinearity distortion correction, realignment of functional images, fieldmap‐based distortion correction, normalization of functional images to an MNI template (the “tissue probability map” [tpm] image in SPM v. 12), and spatial smoothing with a 6‐mm kernel. Registrations of BOLD images to the MNI template were checked manually for each participant as part of our quality control procedures.

### 2.4 Data analysis

#### 2.4.1 Primary model

At the individual subject level, the primary general linear model specified regressors for blink suppression periods based on whether they were early in the period (“Hold1,” the first 30 s) or late in the period (“Hold2,” the last 30 s). Blocks were segregated into early and late periods in order to allow for the differentiation of brain activity based on the buildup of the urge over time, given prior work showing that the urge to blink is greater, on average, during the last half of a 60‐s suppression period than the first half (Botteron et al., 2019, see below for further discussion). Blocks where subjects blinked freely (“Free”) were also modeled (30 s). Regressors for the blink recovery period and the rating period were included to account for variance but were not analyzed further. Six realignment parameters were included to further reduce error variance associated with residual movement after realignment. Additional motion and artifact correction was conducted through spike regression (Ciric et al., 2017). We first identified volumes showing framewise displacement over 2 mm (translation) or 1° (rotation), or global signal over 9*SD* from the mean, using ART (http://www.nitrc.org/projects/artifact_detect) and then regressed these volumes out of the data by specifying them as covariates of no interest in subject‐level models. At the group level, OCD patients and controls were compared on each condition of interest (Hold1, Hold2, Free) using two‐sample *t* tests. Despite sequence harmonization, we statistically controlled for residual differences in image quality between the two scanning locations by specifying site as a covariate for all group‐level imaging analyses, an approach used by multicenter studies including the NIMH‐funded Adolescent Brain Cognitive Development consortium (Glover et al., 2012; Hagler et al., 2019). Unless stated otherwise, stringent correction for false positives used permutation testing (Smith & Nichols, 2009), as suggested by Eklund, Nichols, and Knutsson (2016), and implemented using palm software (Winkler, Ridgway, Webster, Smith, & Nichols, 2014), corrected for multiple comparisons across the whole brain (cluster defining threshold/voxelwise *p* < .005, cluster‐level corrected to family‐wise error [FWE] rate of *p* < .05).

#### 2.4.2 Anatomical parcel localization and post hoc analyses

To determine how the regions that showed group differences mapped onto standard brain atlas parcellation schemes, significant whole‐brain effects (which were found for Hold1 and Hold2 group comparisons, see results) were segregated into regions‐of‐interest (ROIs) based upon the Harvard‐Oxford/Automated Anatomical Labeling (AAL) atlas supplied in the “conn” connectivity toolbox (Whitfield‐Gabrieli & Nieto‐Castanon, 2012). This atlas contains 132 parcels including cortical and subcortical regions (*n* = 106) from the Harvard‐Oxford Atlas (Desikan et al., 2006; Frazier et al., 2005; Goldstein et al., 2007; Makris et al., 2006) plus an additional 26 cerebellar parcels from the AAL atlas (Tzourio‐Mazoyer et al., 2002). We performed conjunction analyses between whole‐brain maps of significant group difference and the 132 parcels to link voxels in the group difference maps to specific anatomical areas of the brain. Parameter estimates from contrasts‐of‐interest were extracted from ROI clusters within the parcels (only those clusters that contained 20 or more contiguous voxels within the parcel were selected) and submitted to post hoc testing. Given the uneven sample sizes between the OCD and control groups, we first tested for unequal variance between the groups in the extracted parameter estimates using Levene's tests. For ROIs where unequal variance was found, degrees of freedom were adjusted using Satterthwaite's approximation. Further post hoc testing of ROI clusters examined whether depressive or anxiety symptoms (as measured by the QIDS or BAI) could explain observed group differences and whether there were effects of medication or psychiatric comorbidity on the findings. All post hoc testing was corrected for comparisons across the multiply tested ROI clusters for each contrast‐of‐interest using false discovery rate (Benjamini & Hochberg, 1995) as implemented in R (p.adjust).

#### 2.4.3 “Urge network” model

To identify the overlap between group differences and a putative network associated with the buildup of the urge to blink, subject‐level contrasts of Hold1 > Free, Hold2 > Free, and Hold2 > Hold1 were analyzed at the group level using one‐sample t‐tests for the entire sample (*n* = 69 subjects, results corrected for multiple comparisons at *p* < .05 using permutation testing as described above). A conjunction analysis probed for regions that were commonly activated for all three pairwise comparisons, reasoning that a region involved in the urge to blink would be more active during both hold blocks (early and late blink suppression) than the free blinking block *and* more active during late blink suppression than early blink suppression as the urge to blink builds over time. Thus, within blink suppression, the buildup of the urge was interrogated by comparing an average of the last 30 s of the blink suppression block to an average of the first 30 s of the block. A different approach was used by Berman et al. (2012), who employed a “sawtooth” model whereby the urge linearly increases from the beginning to the end of the suppression period. Through the analysis of continuous on‐line urge ratings obtained during 60‐s blink suppression blocks, Botteron et al. (2019) showed that an event‐related individualized model—where the urge increases up until there is a blink, after which it reduces somewhat (but not to baseline) before rising again until there is another blink—was superior to the “sawtooth” model because it accounts for temporary reductions in the urge based on accidental blinks occurring during suppression periods. Unfortunately, we could not employ an individualized blink model because we were unable to obtain a reliable measure of eyeblinks for a large portion of participants (discussed in greater detail below). Critically, however, both the sawtooth and individualized blink models predict that, on average, the urge will be greater during the second half than the first half of the suppression period (Berman et al., 2012; Botteron et al., 2019). The present model was selected as a compromise to capture differences between the first and second half of the suppression period without making assumptions about how the urge would vary within each half based on blinks that we were unable to measure.

#### 2.4.4 Exploratory analysis of eyeblink counts

In a secondary analysis, we sought to determine whether there was an effect of block type (eyeblink suppression vs. free blinking) and group (OCD vs. controls) on the total number of blinks, both as a way to confirm participants were following task instructions (there should be fewer eyeblinks during suppression blocks than free blinking blocks) in addition to investigating whether OCD patients showed differential success in suppressing blinks compared to controls. The eye‐tracking system we used identifies blinks as times when the pupil is lost during tracking, and various technical and subject‐specific factors other than real blinking can lead to the loss of the pupil during tracking (e.g., malfunction of eye‐tracking device, subject wearing heavy makeup, subject wearing glasses, subject with eyelids that partially occlude pupil). While we attempted to obtain eyeblink data from all subjects, usable blink data was obtained from only 38 subjects (27 OCD patients and 11 controls). We performed data cleaning to exclude device‐designated blinks that were unlikely to be real blinks. Prior work has shown that the average blink duration is approximately 150–200 ms, with a wide inter‐subject range whose lower end is as brief as 50 ms (Caffier, Erdmann, & Ullsperger, 2003; Wang, Toor, Gautam, & Henson, 2011). We excluded any device‐designated blinks that had durations shorter than 50 ms or longer than 2,000 ms. Finally, we excluded any blinks that occurred during the first 1,000 ms of each new block (both blink suppression and free blinking) to allow participants to adjust to the start of a new screen and instructions. We performed a 2 × 2 mixed analysis of variance (ANOVA) on the total number of blinks in the experiment, with condition (Hold1, Hold2, Free) as within‐subjects factor and group (OCD, control) as between‐subjects factor. Follow‐up independent samples t‐tests were performed to interrogate main effects and interactions.

### 2.5 Results

Clinical and demographic data are shown in Table 1. There were no significant differences between OCD and control groups in age, years of education, or biological sex. As would be expected, OCD patients had increased severity of OC symptoms and SP, as well as greater depression and anxiety symptoms, than controls (see Table 1 for statistical test results).

**Table 1:** Demographic and clinical information

	OCD (*n* = 46)		Controls (*n* = 23)		Group comparison
	Mean *SD*		Mean *SD*		
Age (years)	32.5	11.1	30.3	10.2	*t*(67) = 0.8, *p* = .4
Education (years)	15.9	2.2	16.3	1.8	*t*(67) = 0.7, *p* = .5
Biological sex	32 F/14 M		14 F/9 M		*χ* ^2^(1) = 0.52, *p* = .5
Y‐BOCS (sum)	24.6	5.2	0.28	1.4	*t*(55.9) = 29.7*, *p* < .001
SPS (sum)	8.0	3.2	0.24	0.8	*t*(55.0) = 15.5*, *p* < .001
QIDS (av)	0.73	0.46	0.15	0.18	*t*(64.7) = 7.4*, *p* < .001
BAI (av)	0.80	0.52	0.07	0.14	*t*(56.3) = 8.9*, *p* < .001

*Note*: *Levene's tests for equality of variances revealed unequal variance between the groups for Y‐BOCS, SPS, QIDS, and BAI scores; degrees of freedom for these tests are adjusted using Satterthwaite's approximation. Scores for the Y‐BOCS and SPS reflect the sum of individual rating scales; scores for the QIDS and BAI are the average (av) of responses to the individual questions (total average scores can range from 0 to 3).

Abbreviations: BAI, Beck Anxiety Inventory; OCD, obsessive–compulsive disorder; QIDS, quick inventory of depressive symptoms; *SD*, standard deviation; SPS, sensory phenomena scale; Y‐BOCS, Yale‐Brown Obsessive–Compulsive Scale.

#### 2.5.1 Group differences in brain activation during blink suppression

**Figure 1:**
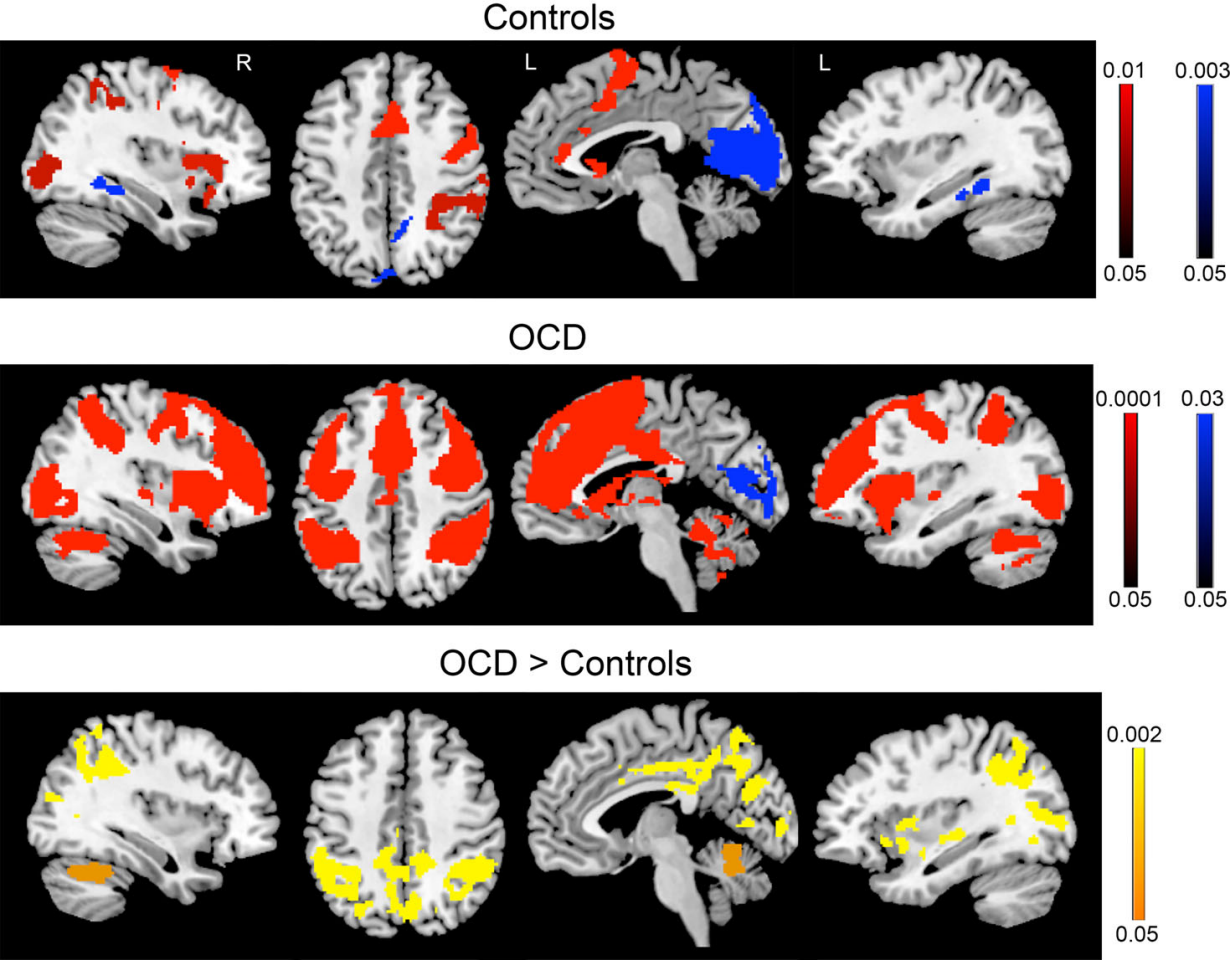
Activity in obsessive–compulsive disorder (OCD) patients and controls during early eyeblink suppression. Controls (top panel) and OCD patients (middle panel) for the comparison of early eyeblink suppression > free blinking (red) and free blinking > early eyeblink suppression (blue). Group differences (bottom panel) revealed increased activity in patients compared to controls in the insula, cingulate cortex, inferior parietal cortex, occipital regions, and cerebellum. No significant differences were found for free blinking blocks. Color bars represent whole‐brain family wise error corrected *p* value

During early blink suppression (Hold1), OCD patients showed significantly greater activity than controls in three large clusters (Figure 1, bottom panel). The largest cluster (*k* = 10,470, whole‐brain corrected *p* value = .002) was composed of mostly posterior brain regions and included anterior, mid, and posterior cingulate, postcentral gyrus, inferior and superior parietal cortex and precuneus, lateral, and medial occipital regions, and parahippocampal gyrus. The second cluster (*k* = 565, whole‐brain corrected *p* value = .002) contained voxels located in anterior and mid‐insula (BA 13, 47), inferior frontal gyrus (BA 47), claustrum, caudate, putamen, amygdala, and parahippocampal gyrus. The third cluster was in the cerebellum (*k* = 1,048, whole‐brain corrected *p* value = .04), predominantly in the culmen and declive portions. There were 47 ROIs where atlas parcels contained 20 or more voxels from the OCD > controls comparison for the Hold1 contrast (Table 2). Levene's tests performed on the parameter estimates indicated that variances were not statistically unequal between the groups for any ROI. For one of the insula ROIs (*k* = 42) and one of the putamen ROIs (*k* = 123), there were trends toward unequal variances between the groups (Levene's test *F* = 3.92, *p* = .052, and *F* = 3.43, *p* = .069, respectively). However, *t* tests comparing OCD and controls for these ROIs when adjusting degrees of freedom using Satterthwaite's approximation remained highly significant (*t*(61.92) = 3.74, *p* < .001 and *t*(62.37) = 4.13, *p* < .001, respectively).

**Table 2:** Greater activation in OCD patients than controls during early eyeblink suppression

Label	Harvard‐Oxford parcel	BA	*k*	*x*	*y*	*z*
*Frontal*/*insular*						
Inferior frontal gyrus/orbital gyrus	Frontal orbital cortex (L)	47	20	−42	18	−8
Insula/inferior frontal gyrus	Insular cortex (L)	13, 47	126	−36	6	−12
Insula/claustrum	Insular cortex (L)		42	−32	12	0
Mid cingulate/anterior cingulate	Cingulate gyrus, anterior (L)	24, 32	80	−6	10	30
*Parietal*						
Paracentral lobule/precuneus/postcentral gyrus	Postcentral gyrus (R)	4, 5	28	14	−42	56
Inferior parietal lobule/supramarginal gyrus/angular gyrus	Angular gyrus (L)	40	246	−50	−56	26
Inferior parietal lobule/supramarginal gyrus/angular gyrus	Angular gyrus (R)	40	315	54	−44	24
Inferior parietal lobule/superior parietal lobule	Superior parietal lobule (L)	7, 40	178	−32	−54	38
Inferior parietal lobule/superior parietal lobule	Superior parietal lobule (R)	7, 40	218	32	−46	38
Inferior parietal lobule/supramarginal gyrus	Supramarginal gyrus, anterior (L)	40	128	−60	−38	36
Inferior parietal lobule/supramarginal gyrus	Supramarginal gyrus, posterior (L)	40	323	−58	−52	30
Inferior parietal lobule/supramarginal gyrus/postcentral gyrus	Supramarginal gyrus, posterior (R)	40	464	52	−44	22
Superior parietal lobule/middle occipital gyrus/precuneus/inferior parietal lobule/angular gyrus	Lateral occipital cortex, superior (L)	7, 19, 39, 40	620	−22	−82	20
Angular gyrus/superior parietal lobule/inferior parietal lobule/precuneus/middle occipital gyrus	Lateral occipital cortex, superior (R)	7, 39, 40	337	42	−62	30
Mid cingulate/posterior cingulate/precuneus	Cingulate gyrus, posterior (B)	23, 24, 31	555	−2	−38	24
Posterior cingulate/precuneus/lingual gyrus	Cingulate gyrus, posterior (B)	29	28	22	−48	2
Precuneus/cuneus/mid cingulate/posterior cingulate/superior parietal lobule/paracentral lobule	Precuneus cortex (B)	5, 7, 20, 31	1,702	10	−54	4
Calcarine/posterior cingulate	Precuneus cortex (L)	30	20	−20	−62	4
*Occipital*						
Calcarine/cuneus/posterior cingulate/lingual gyrus	Intracalcarine cortex (L)	17, 18, 23, 30	173	−16	−68	2
Calcarine/cuneus/posterior cingulate/lingual gyrus	Intracalcarine cortex (R)	30	72	22	−64	2
Cuneus/calcarine/lingual gyrus	Intracalcarine cortex (R)	30	53	6	−84	0
Calcarine/posterior cingulate/cuneus	Supracalcarine cortex (R)	31	41	22	−64	12
Cuneus	Supracalcarine cortex (R)	17, 18	21	2	−86	4
Lingual gyrus/cuneus/parahippocampal gyrus/precuneus	Lingual gyrus (L)	18, 19, 30	120	−16	−48	−4
Lingual gyrus/cerebellum	Lingual gyrus (L)		67	−18	−66	−14
Lingual gyrus/calcarine/cuneus	Lingual gyrus (R)	18, 30	119	10	−88	−12
Lingual gyrus/calcarine/parahippocampal gyrus	Lingual gyrus (R)	18, 19, 30	56	20	−76	−4
Cuneus/precuneus/superior occipital gyrus	Cuneal cortex (L)	7, 18, 19, 31	198	−14	−74	18
Cuneus/precuneus	Cuneal cortex (R)	7, 18, 19, 31	389	2	−88	14
Middle occipital gyrus/inferior occipital gyrus/inferior temporal gyrus	Lateral occipital cortex, inferior (L)	19, 37, 39	316	−42	−70	−12
Middle occipital gyrus	Lateral occipital cortex, inferior (R)	19	31	36	−82	6
Middle occipital gyrus/middle temporal gyrus	Lateral occipital cortex, superior (L)	19	83	−34	−88	6
Middle occipital gyrus/ middle temporal gyrus	Lateral occipital cortex, superior (R)	19	85	38	−82	10
Middle occipital gyrus	Lateral occipital cortex, superior (R)	19	53	28	−72	22
Cuneus/middle occipital gyrus/superior occipital gyrus/calcarine	Occipital pole (L)	17, 18, 19	265	−4	−92	−2
Cuneus/calcarine	Occipital pole (R)	18, 19	29	4	−90	4
*Subcortical*						
Caudate head	Caudate (L)		44	−14	16	−4
Putamen (lentiform nucleus)	Putamen (L)		123	−26	4	−10
Putamen (lentiform nucleus)	Putamen (L)		29	−32	−16	−8
Hippocampus/thalamus	Thalamus (L)	27	31	−20	−34	−4
Hippocampus/parahippocampal gyrus	Hippocampus (L)	27, 30	73	−24	−30	−12
Cerebellum (culmen/declive)	Cerebellum crus 1 (R)		181	42	−52	−36
Cerebellum (culmen/declive)	Cerebellum 4_5 (L)		49	−10	−56	−22
Cerebellum (culmen/declive)	Cerebellum 6 (L)		217	−14	−64	−28
Cerebellum (culmen/declive)	Cerebellum 6 (R)		345	30	−56	−34
Cerebellum (declive)	Vermis 6 (B)		51	−4	−62	−24
Cerebellum (declive)	Vermis 7 (B)		27	6	−66	−26

*Note*: Labels are derived from the AAL and Talairach Daemon databases as provided through xjview (v. 9.6, http://www.alivelearn.net/xjview). Harvard‐Oxford Atlas parcels are provided through the “conn” tool (Whitfield‐Gabrieli & Nieto‐Castanon, 2012). Within a given cluster, labels are listed in descending order based on the proportion of voxels within the cluster assigned to that label. Some clusters span across two different lobes; for these clusters, lobe assignment is based on the lobe with the greatest proportion of voxels in that cluster. Coordinates are in MNI space. Only clusters with 20 or more contiguous voxels are listed. Parcels represent subdivisions from clusters corrected for a whole‐brain FWE rate of *p* < .05 using permutation testing.

Abbreviations: AAL, Automated Anatomical Labeling; B, bilateral; BA, Brodmann's areas; FWE, family wise error; *k*, cluster extent; L, left; OCD, obsessive–compulsive disorder; R, right.

**Figure 2:**
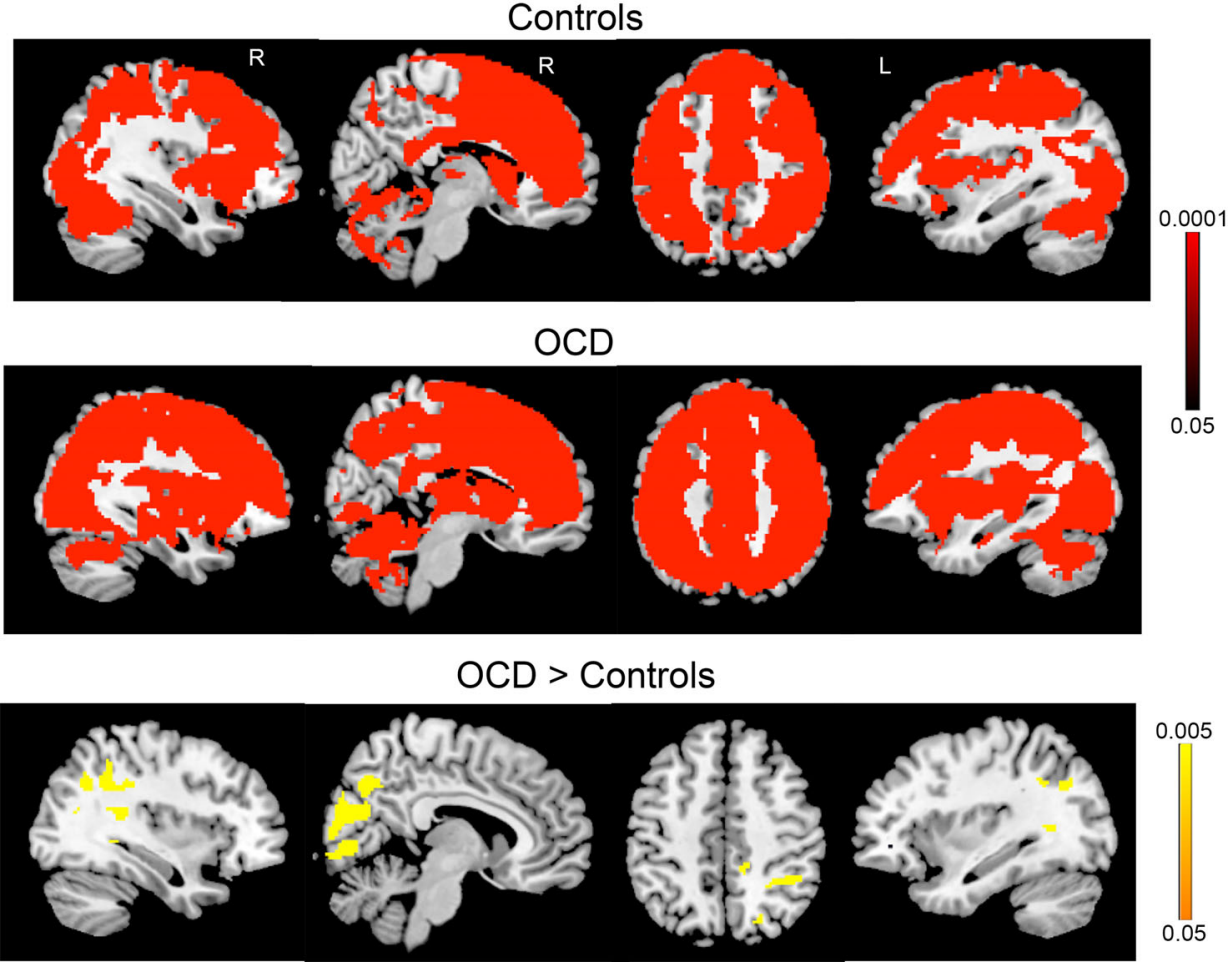
Activity in obsessive–compulsive disorder (OCD) patients and controls during late eyeblink suppression. Controls (top panel) and OCD patients (middle panel) for the comparison of late eyeblink suppression > free blinking (red). There were no areas where free blinking > late eyeblink suppression for either group. Group differences (bottom panel) revealed increased activity in patients compared to controls in inferior parietal cortex and occipital regions. No significant differences were found for free blinking blocks. Color bars represent whole‐brain family wise error corrected *p* value

During late eyeblink suppression (Hold2), OCD patients showed greater activity than controls in two clusters in occipital and parietal cortex (Figure 2, bottom panel). The first large cluster (*k* = 4,631, whole‐brain corrected *p* value = .005) included bilateral medial occipital cortex (BAs 17, 18, 19, 30), precuneus and posterior cingulate (7, 23, 31), right inferior parietal cortex (BA 40), and right parahippocampal gyrus. The second cluster (*k* = 246, whole‐brain corrected *p* value = .005) included left hemisphere areas of precuneus and medial occipital cortex (BA 7) and inferior parietal cortex (BA 40). There were 22 ROIs where atlas parcels contained 20 or more voxels identified from the OCD > controls comparison for the Hold2 contrast (Table 3). Levene's tests performed on the parameter estimates indicated that variances were not statistically unequal between the groups for any ROI. For the left occipital pole ROI (*k* = 251), there was a trend toward unequal variance between the groups (Levene's test *F* = 3.01, *p* = .087). However, the *t* test comparing OCD and controls for this ROI when adjusting degrees of freedom using Satterthwaite's approximation remained significant (*t*(32.20) = 3.56, *p* = .001).

**Table 3:** Greater activation in OCD patients than controls during late eyeblink suppression

Label	Harvard‐Oxford parcel	BA	*k*	*x*	*y*	*z*
*Parietal*						
Inferior parietal lobule	Superior parietal lobule (R)	40	63	32	−46	38
Mid cingulate	Cingulate gyrus, posterior (R)	31	32	14	−46	36
Precuneus/cuneus	Precuneus cortex (R)	31	106	14	−62	16
Precuneus/mid cingulate	Precuneus cortex (R)	31	43	14	−46	40
Calcarine/lingual gyrus/posterior cingulate	Precuneus cortex (R)	29	23	10	−54	4
Precuneus	Precuneus cortex (L)	7	22	−16	−72	34
Precuneus/cuneus	Precuneus cortex (R)	7	234	16	−70	28
*Occipital*						
Calcarine/cuneus/posterior cingulate	Intracalcarine cortex (L)	17, 18, 23, 30	225	−16	−68	2
Cuneus	Intracalcarine cortex (L)	17, 18	31	−2	−90	−4
Calcarine/cuneus/posterior cingulate	Intracalcarine cortex (R)	17, 18, 30	289	8	−84	0
Calcarine/posterior cingulate	Supracalcarine cortex (R)	31	39	22	−64	12
Cuneus/calcarine	Supracalcarine cortex (R)	18, 31	30	4	−88	8
Cuneus/precuneus	Cuneal cortex (L)	7, 18, 19, 31	228	−14	−76	18
Cuneus/precuneus	Cuneal cortex (R)	7, 18, 19, 31	360	2	−88	14
Lingual gyrus/cuneus/parahippocampal gyrus	Lingual gyrus (L)	18, 30	114	−32	−42	−8
Lingual gyrus/calcarine	Lingual gyrus (L)	18	29	−4	−86	−14
Lingual gyrus/calcarine	Lingual gyrus (R)	18	162	10	−90	−12
Lingual gyrus/parahippocampal gyrus	Lingual gyrus (R)	18, 19, 30	93	16	−50	−4
Middle occipital gyrus/precuneus/superior occipital gyrus	Lateral occipital cortex, superior (L)	7, 39	96	−26	−66	30
Precuneus/superior occipital gyrus/middle occipital gyrus/angular gyrus	Lateral occipital cortex, superior (R)	7, 39	197	28	−72	24
Cuneus/middle occipital gyrus	Occipital pole (L)	17, 18, 19	251	−4	−92	−2
Cuneus/calcarine/middle occipital gyrus	Occipital pole (R)	18	41	2	−90	4

*Note*: Labels are derived from the AAL and Talairach Daemon databases as provided through xjview (v. 9.6, http://www.alivelearn.net/xjview). Harvard‐Oxford Atlas parcels are provided through the “conn” tool (Whitfield‐Gabrieli & Nieto‐Castanon, 2012). Within a given cluster, labels are listed in descending order based on the proportion of voxels within the cluster assigned to that label. Some clusters span across two different lobes; for these clusters, lobe assignment is based on the lobe with the greatest proportion of voxels in that cluster. Coordinates are in MNI space. Only clusters with 20 or more contiguous voxels are listed. Parcels represent subdivisions from clusters corrected for a whole‐brain FWE rate of *p* < .05 using permutation testing.

Abbreviations: Abbreviations: AAL, Automated Anatomical Labeling; B, bilateral; BA, Brodmann's areas; FWE, family wise error; *k*, cluster extent; L, left; OCD, obsessive–compulsive disorder; R, right.

There were no areas where controls showed significantly greater activity than OCD patients for either early or late blink suppression, and there were no significant group differences between OCD and controls during the free blinking condition.

#### 2.5.2 Activation associated with buildup of the urge to blink

**Figure 3:**
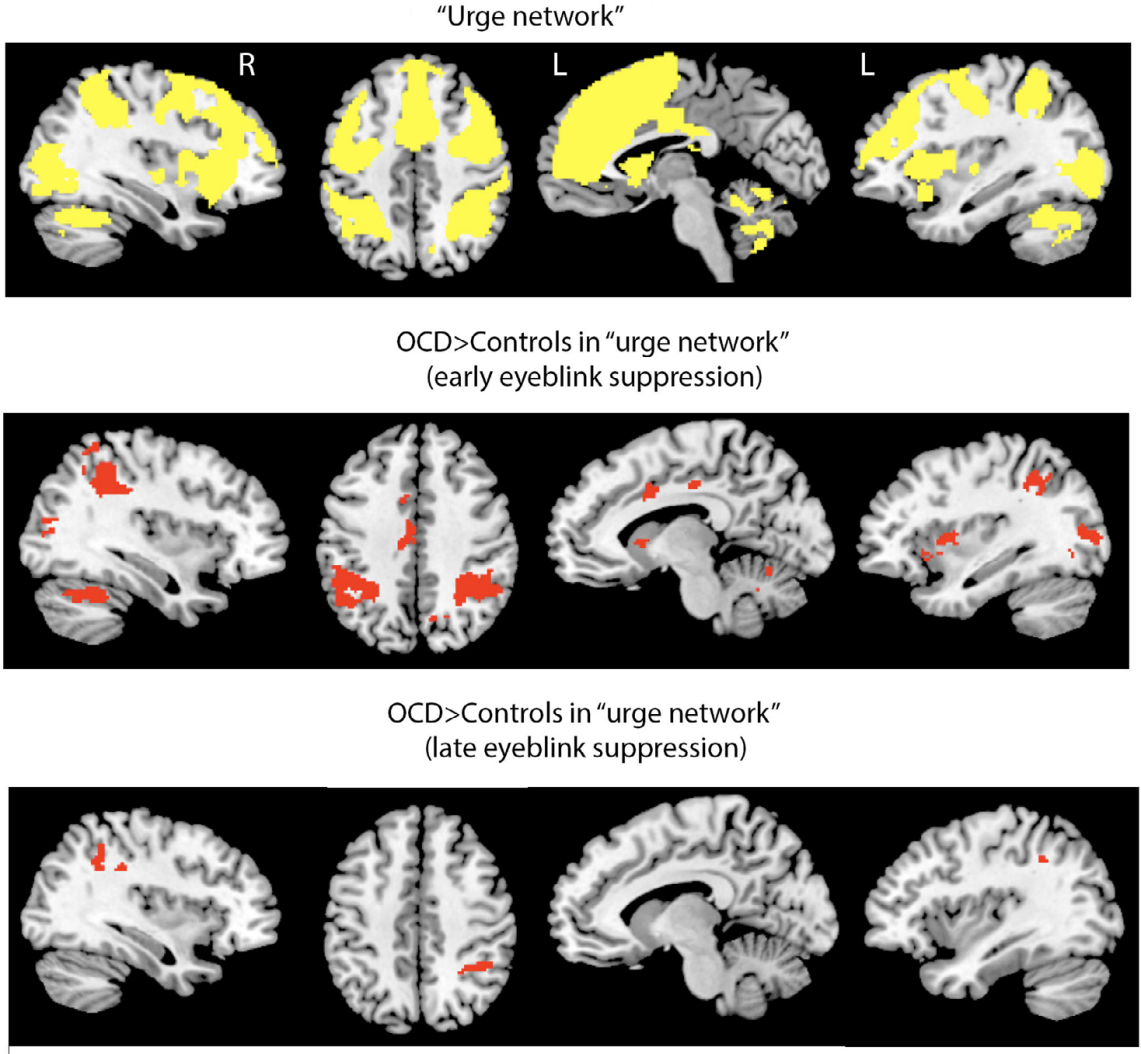
Putative “urge network.” Areas where early eyeblink suppression > free blinking, late eyeblink suppression > free blinking, and late > early eyeblink suppression in the full sample (top panel). In order to aid in the interpretation of group differences, the middle and bottom panels show the overlap between areas showing differences between obsessive–compulsive disorder (OCD) patients and controls for early and late eyeblink suppression, respectively, and regions in the “urge network”

To identify the overlap between the areas reported above showing group differences and a putative network associated with the buildup of the urge to blink, we performed a conjunction analysis to identify those regions where Hold1 > Free, Hold2 > Free, and Hold2 > Hold1 for the entire sample of subjects, irrespective of group membership (whole‐brain maps showing Hold1 > Free and Hold2 > Free for each group separately are shown in Figures 1 and 2). This analysis revealed widespread areas of the cortex and subcortex that were significantly activated in all three comparisons (Figure 3, top panel). These activations separated into two major clusters, one very large cluster (*k* = 50,152) consisting of dorsolateral and ventrolateral prefrontal cortex extending into temporal pole (BA 8, 9, 38, 44, 45, 46, 47), frontal pole (BA 10), anterior and mid‐cingulate and SMA (BA 6, 23, 24, 32, 33), precentral and postcentral gyri (BAs 2, 3, 4, 5, 6, 43), inferior and superior parietal cortex (BAs 7, 39, 40), insula (BA 13, 47), posterior temporal cortex (BAs 20, 21, 22, 41, 42), lateral occipital cortex (BA 18, 19, 37), parahippocampus, caudate, putamen, thalamus, and claustrum. The other significant cluster was located in the cerebellum (*k* = 3,351) and consisted of areas of the declive, culmen, uvula, tuber, and nodule.

To aid in the interpretation of group differences, we sought to determine if any of those areas associated with this putative “urge network” overlapped with regions showing differences between OCD patients and controls. Out of the areas showing increased activity for OCD patients compared to controls for early eyeblink suppression (Hold1), cingulate cortex, insula, putamen, caudate, superior and inferior parietal cortex, precuneus, lateral occipital cortex, and cerebellum overlapped with the “urge network” (Figure 3, middle panel). Out of those areas showing increased activity in OCD for late eyeblink suppression (Hold2), lateral occipital cortex, inferior parietal cortex, and precuneus overlap with the “urge network” (Figure 3, bottom panel).

#### 2.5.3 Controlling for depression and anxiety

Parameter estimates from ROIs listed in Tables 2 and 3 were submitted to separate one‐way ANOVAs with group (OCD vs. HC) as independent variable and either QIDS score (for depression severity) or BAI score (for anxiety severity) as covariates, to determine if OCD patients still showed significantly greater activity than controls when statistically controlling for depression or anxiety symptoms (false discovery rate (FDR) corrected *p* values shown in Supplemental Tables S1 and S2). Parameter estimates for all ROIs remained significantly greater in OCD than controls when controlling for depression severity. When controlling for anxiety severity, all ROI estimates remained significantly higher in OCD except the lateral occipital cortex, superior (right hemisphere) cluster, which was different from controls at trend level (*p* = .055, see Supplemental Table S1). These data indicate that the group differences found during blink suppression were not driven by differences in depression or anxiety symptoms between the groups.

#### 2.5.4 Effects of medication and comorbidities

Within the OCD group, there were no significant differences between unmedicated OCD patients (*n* = 22) and medicated OCD patients (*n* = 24), or between OCD patients without any comorbidities (*n* = 15) and patients with one or more comorbid disorders (*n* = 31), in ROI parameter estimates for either the Hold1 or Hold2 contrast (Tables 2 and 3). Independent samples *t* tests confirmed that unmedicated OCD patients showed significantly elevated activity in all ROIs listed in Tables 2 and 3 compared to controls (FDR corrected *p* values shown in Supplemental Tables S1 and S2). In addition, OCD patients without any comorbidity showed increased activation in all ROIs, with the exception of the cerebellum 4_5 (left hemisphere), superior parietal lobule (right hemisphere), and lateral occipital cortex, superior (left hemisphere) ROIs, which showed trend‐level increases relative to controls (FDR‐corrected *p* values of .067, .080, and .070, respectively; see Supplemental Tables S1 and S2). These analyses indicate that neither medication nor comorbidity in the OCD cohort was driving the differences identified between patients and controls.

#### 2.5.5 Relationship between brain activity and symptoms

Within the OCD group, none of the ROIs listed in Tables 2 and 3 were significantly related to overall obsessive–compulsive symptom severity (as measured by the Y‐BOCS) after correcting for multiple comparisons using FDR. Contrary to predictions, none of the ROIs were significantly related to SP severity (as measured by the SPS) either.

Given our prior study identifying a positive correlation between SP severity and activity in the insula and somatosensory cortex using a different fMRI task (Brown et al., 2019), we sought to investigate whether a search specifically within those regions would reveal any relationships with SPS score even if the ROIs identified from our group comparisons did not. Searching within the mask of insula and sensorimotor regions used in that prior study (see Brown et al., 2019, composed of insula, precentral and postcentral gryi, SMA, and paracentral lobule), there were no clusters whose activity was correlated with SPS score for any of the three conditions‐of‐interest (Hold1, Hold2, Free) after correcting for multiple comparisons (FWE rate of *p* < .05 using permutation testing). However, for early eyeblink suppression (Hold1), there was a correlation between SPS score and activity in cluster located in left precentral/postcentral gyrus (*k* = 183, *x* = −60, *y* = −8, *z* = 22, BAs 3, 4, and 6) that was trend‐level significant (FWE corrected *p* value = .076). When further probing the Hold1 condition at an uncorrected threshold (voxelwise *p* < .005, *k* = 20), we found eight clusters within the mask where greater activity was associated with increased severity of SP, including bilateral mid/posterior insula, bilateral precentral/postcentral gyri, and paracentral lobule (uncorrected *p* values ranging from .0013 to .0008, see Supplemental Table S3). Although these data indicate that certain areas in the UFA network did show a correlation with SP severity, the findings must be interpreted with caution and require replication given that they did not survive correction for multiple comparisons.

### 2.6 Eyeblink counts

**Figure 4:**
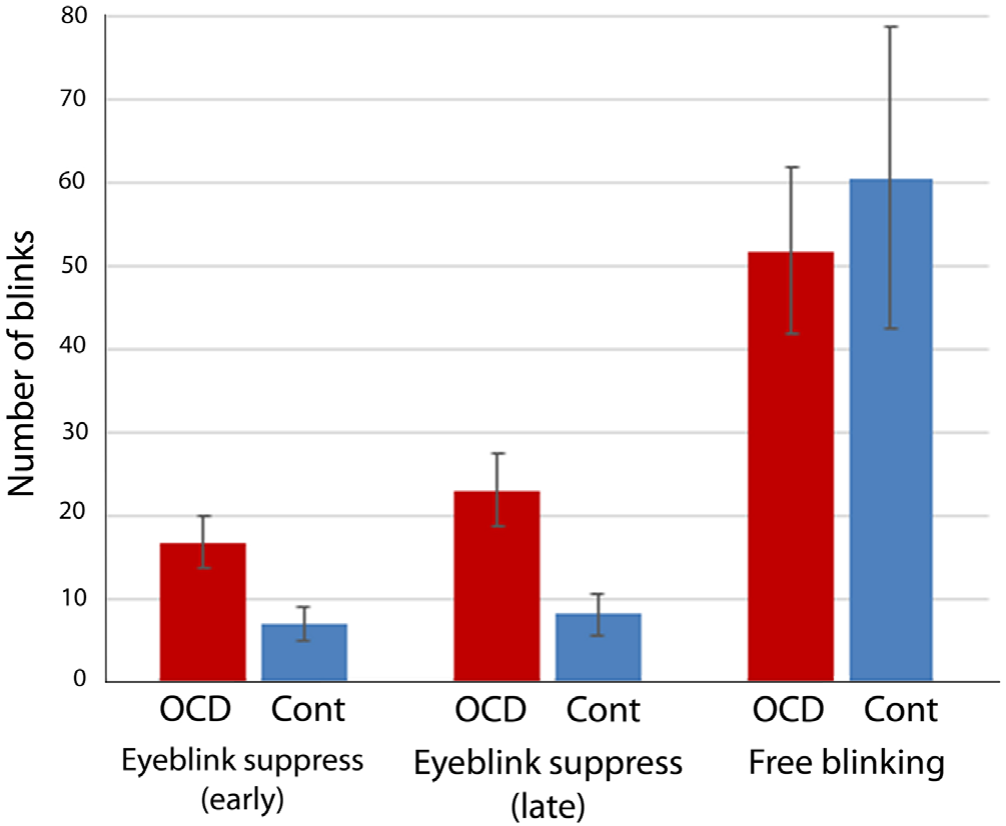
Blink counts in a subset of participants. Obsessive–compulsive disorder (OCD) patients (red bars) blinked significantly more than controls (blue bars) during early and late eyeblink suppression, but were not different during free blinking

In an exploratory analysis of total eyeblink counts conducted in a subset of subjects (27 OCD patients and 11 controls), the distributions of blink counts for Hold1, Hold2, and Free blocks were not significantly different from normal for either OCD or control groups (0.20 > *p* > .08 for all). Levene's tests indicated that variances were not statistically unequal between the groups for any of the three conditions despite the unequal sample sizes. A 2 × 2 ANOVA with block type (Hold1, Hold2, Free) and group (OCD, controls) as factors revealed a main effect of block type (*F* (1.1,40.3) = 105.38, *p* < .001, degrees of freedom adjusted for violations of sphericity using Greenhouse–Geisser correction). Follow‐up paired *t* tests revealed significant differences between all block types in blink counts, with fewer total blinks for Hold1 (*n* = 13.8) than Free (*n* = 54.2) (*t*(37) = 10.38, *p* < .001); fewer blinks for Hold2 (*n* = 18.6) than Free (*t*(37) = 8.79, *p* < .001); and fewer blinks for Hold1 than Hold2 (*t*(37) = 4.38, *p* < .001). There was also an interaction between block type and group (*F*(1.1,40.3) = 6.77, *p* = .011) such that OCD patients showed significantly more blinks than controls during Hold1 (*t*(36) = 2.17, *p* = .036, OCD: 16.7 [rate of 2.09 blinks per 30‐s period], controls: 6.7 [rate of 0.84 blinks per 30‐s period]) and Hold2 (*t*(36) = 2.89, *p* = .006, OCD: 22.9 [rate of 2.87 blinks per 30‐s period], controls: 8.0 [rate of 1 blink per 30‐s period]), but were not statistically different from controls for Free blinking (*t*(36) = −0.97, *p* = .338, OCD: 51.7 [rate of 6.46 blinks per 30‐s period], controls: 60.5 [rate of 7.56 blinks per 30‐s period]) (Figure 4). These data provide evidence that both OCD patients and controls were able to comply with task demands and were generally successful at suppressing eyeblinks during suppression blocks. They also indicate that OCD patients experienced greater difficulty suppressing eyeblinks during early and late phases of the suppression period, as evidenced by the increased number of blinks for the patient group.

### 2.7 Urge ratings

Analysis of the ratings of urge intensity that subjects made after each 60‐s blink suppression period revealed that, on average, the sample rated the suppression period as eliciting a moderate‐to‐strong urge (on a scale of 1–5, mean rating across all subjects: 3.79, mean rating of OCD patients: 3.87, mean rating of controls: 3.64). The distribution of mean urge ratings was significantly different from normal in the OCD group (Kolmogorov–Smirnov [K–S] test of normality = 0.167, *p* = .003), with moderate negative skewness (−0.883). By contrast, the distribution of the control group's ratings was not significantly different from normal (K–S test = 0.112, *p* = .2). A nonparametric independent samples Mann–Whitney *U* test did not reveal a significant difference in the distributions of mean urge ratings between the groups (*p* = .2). However, when examining the proportion of participants whose urge rating fell above the sample mean, 63.0% of OCD patients (29/46) and 39.1% of controls (9/23) had average ratings higher than the mean (*χ* ^2^ = 3.5, *p* = .06), revealing a trend toward there being a greater proportion of OCD patients than controls reporting more intense urges to blink during suppression periods. Supplemental Figure S1 shows the distribution of mean urge ratings for each group.

### 2.8 Discussion

We used an eyeblink suppression task in OCD patients and controls in order to examine neural mechanisms underlying the buildup and suppression of an UFA. During early eyeblink suppression (the first 30 s), OCD patients showed significantly greater activity than controls in a set of brain regions including parietal cortex, occipital cortex, posterior and anterior cingulate, insula, caudate, putamen, thalamus, hippocampus, and cerebellum. During late eyeblink suppression (the last 30 s), parietal and occipital regions remained hyperactive in the OCD group. Post hoc analyses indicated that group differences were not due to effects of medication or comorbidities in the OCD group, as subgroups of unmedicated patients and patients without comorbidities also showed elevated activity in these areas compared to controls. Furthermore, group differences remained significant after statistically controlling for depression and anxiety symptoms. In an exploratory analysis on the subset of subjects for which we had eyeblink measurements, OCD patients were less successful than controls in suppressing blinks during the blink suppression blocks but no different in overall blinking rate during the free blinking blocks. Although this eyeblink count analysis was conducted only in a subset of participants, a similar difference has been reported between patients with TD and controls (Botteron et al., 2019), lending credence to the findings. Overall, these data indicate that OCD patients exhibit altered brain function and behavior when experiencing and suppressing the urge to blink. Given that these urges are unrelated to OCD symptoms, this finding raises the possibility that the disorder is associated with a more general abnormality in the UFA system that could ultimately be targeted by future treatments.

Our hypothesis that OCD patients would show hyperactivity during blink suppression in regions previously shown to be related to UFA—namely, insula and sensorimotor cortical regions including precentral and postcentral gyri and cingulate—was partially confirmed. OCD patients showed hyperactivity of mid and anterior regions of insula and mid‐cingulate cortex during early eyeblink suppression. The insula and mid‐cingulate (sometimes referred to as the cingulate motor area) are considered key nodes of the UFA network, showing common activation for various urges including the urge to yawn, studies of swallowing and micturition, and the urge to tic (Jackson et al., 2011). Multiple lines of research from brain imaging, stimulation, and lesion studies suggest that the insula in particular plays a fundamental role in interoception and the processing of sensation from within the body (Aziz et al., 1997; Craig, 2002, 2003; Critchley & Harrison, 2013; Eickhoff et al., 2006; Ibanez, Gleichgerrcht, & Manes, 2010; Isnard, Guenot, Sindou, & Mauguiere, 2004; Ostrowsky et al., 2002); as such, insula hyperactivity in OCD may reflect a heightened experience of the physical sensation of the urge during the early part of the blink suppression period in OCD.

Contrary to predictions, however, other sensorimotor cortical regions such as precentral and postcentral gyri were not prominently hyperactive in OCD during blink suppression, despite being associated with UFA both in prior work (Berman et al., 2012; Bohlhalter et al., 2006; Holle et al., 2012; Lerner et al., 2009; Mazzone et al., 2011; Neuner et al., 2014) and in the present study as part of our “urge network” analysis. Instead, activation in more posterior parietal areas extending into occipital cortex was increased in OCD during early and late eyeblink suppression. Although posterior parietal and occipital areas are not typically associated with UFA (Jackson et al., 2011), several of these areas were a part of our “urge network” analysis, suggesting that the brain regions associated with eyeblink suppression may be more widespread than those associated with the suppression of other types of urges. Indeed, Berman et al. (2012) also found posterior parietal (inferior parietal lobule/supramarginal gyrus) and occipital activity associated with the build‐up of the urge to blink. Posterior parietal cortex is considered a multimodal association area, receiving input from visual, somatosensory, motor, cingulate, and prefrontal regions (Whitlock, 2017), and linked to a diverse array of processes including sensorimotor integration, spatial and sustained attention, and higher order cognitive functions (Ptak, 2012; Smith et al., 2009; Whitlock, 2017). Particularly relevant for the present study, posterior parietal cortex interacts with nearby occipital regions by sending top‐down signals to bias visual processing for attended stimuli (Lauritzen, D'Esposito, Heeger, & Silver, 2009; Silvanto, Muggleton, Lavie, & Walsh, 2009; Whitlock, 2017). One can speculate that the simultaneous activation of parietal and occipital regions during blink suppression represents the engagement of visuospatial attentional processes in an attempt to prevent blinking. Given that the blink count analysis showed that OCD patients were actually less successful in suppressing blinking than controls, the increased recruitment of visuospatial attentional regions in OCD may track attentional effort or difficulty rather than being an effective strategy for suppression.

It is important to note that, in our task, the suppression blocks would be expected to engage neural areas involved in the experience of the urge to blink as well as those responsible for preventing an individual from acting on that urge. Botteron et al. (2019) recently showed that discomfort associated with the urge to blink was almost completely collinear with the amount of effort required to suppress blinks. In the current observational study, there is no way to disentangle these two processes and we must acknowledge that the brain activations we identified in the “urge network” likely reflect both the increasing urge (i.e., increasing discomfort) over time as well as the increasing effort required to continually suppress the blink in the face of the increasing urge. Future work using intervention methods such as brain stimulation to modulate different nodes of the network could help to distinguish these regions, as inhibition of an area involved in actively suppressing blinking would be expected to have an opposite effect on suppression success than the inhibition of a region involved in the experience of the discomfort associated with the urge. It is interesting, however, that we did not find any regions where OCD patients showed *less* activation than controls during blink suppression. This is in contrast to findings in TD relating greater tic severity to reduced activity during blink suppression in inferior frontal cortex, superior temporal gyrus, and putamen, presumably reflecting reduced inhibitory control in patients with more severe tics (Mazzone et al., 2010). Unlike in that study, the present results provide no evidence of a hypoactive inhibitory system associated with deficient blink suppression in OCD.

The majority of the neural differences between OCD patients and controls occurred during the early blink suppression period, with fewer lingering group differences at the late suppression period. Indeed, the late suppression period was associated with strong and widespread activations in both OCD and control groups (see Figure 2), as might be expected given the increasing urge and difficulty in suppression as the 60‐s period elapsed. Many of those areas that showed hyperactivity in OCD during the early suppression period were engaged by both groups during late blink suppression. These data suggest that rather than there being a fundamental difference between patients and controls in how the brain experiences and suppresses urges, there may be a difference in the timing of the brain's urge response, with OCD patients experiencing a stronger response earlier than controls. This finding suggests that a thorough understanding of how neural mechanisms of urges and urge suppression play a role in OCD must consider not only how strongly the network is activated, but also how quickly it is activated when the need to suppress a behavior arises. As the urge ratings we obtained during the task did not distinguish between early and late phases of the suppression period, we do not have behavioral data that can address this issue. However, prior work has identified differences in the time courses of urges to perform mental compulsions in OCD and urges to blink in healthy controls (Brandt et al., 2018). We also cannot rule out the possibility that there was increased variability of mental state or task engagement during late blink suppression (as compared to early blink suppression), due to its greater distance from the onset of the blink suppression period, that could have reduced power to detect a group difference during this later time period.

None of the areas that were hyperactive in OCD were significantly related to general OCD symptom severity (as measured by the Y‐BOCS) or specifically to SP severity. This was contrary to our predictions as SP are characterized by physical sensations and urges (Ferrao et al., 2012; Lee et al., 2009; Rosario et al., 2009; Shavitt et al., 2014), and in prior work we found that a region of mid‐insula (in an area overlapping with the area of the insula that was hyperactive in the present study) was related to SP severity (Brown et al., 2019). At lower thresholds, we did identify correlations between SP severity and insula and sensorimotor activity in OCD during early eyeblink suppression, yet all but one of these findings did not surpass corrections for multiple comparisons. It is unclear why the relationships between SP and urge‐related activations were rather weak in the present study; we can speculate that the task itself, which is designed to induce a physical urge in all participants (both patients and controls), may have engaged urge‐related circuits to such an extent that more subtle effects related to endogenous differences between patients with and without SP may have been obscured.

To our knowledge, this is the first study to investigate the neural correlates of urges in OCD, with neural and behavioral results providing insight into the pathophysiology of an understudied and important aspect of the disorder. However, there are several limitations of the current work, which suggest avenues for future research. As described earlier, a good model of the urge to blink is one where the urge rises and falls in accordance with specific blink events during suppression periods (Botteron et al., 2019), yet we could not specify this type of model because we did not obtain reliable eyeblink measurements on all participants. In the future, we will seek to obtain improved eyeblink data, such as through videotaping of the eye (see Botteron et al., 2019) or electrooculogram (Denney & Denney, 1984), or obtain continuous urge ratings (Botteron et al., 2019) rather than ratings occurring only at the end of the suppression period. Another issue to consider is that our suppression blocks often included some accidental blinks (depending on how successful an individual was in suppressing blinking), which may be contributing to the BOLD signal measured during these blocks. However, we believe it is unlikely that the group differences found during blink suppression were related to differences between patients and controls in the number of blinks (a finding identified in the subset analysis but not confirmed for the full sample), as there is little evidence that a higher blink rate is associated with greater activity in the identified regions. For example, a comparison of free blinking blocks with late suppression blocks reveals no areas with greater activation for free blinking (Figure 2), despite there being the expected higher blink rate for free than late suppression blocks in both groups in the subset analysis. This suggests that the increased activity in OCD patients during suppression periods is not likely to be due to higher blinking rate in the patient group.

We compared the buildup of an urge between OCD patients and controls using a blink suppression task in both groups, similar to other work in TD (Mazzone et al., 2010), which has the advantage of allowing us to compare brain function and behavior in response to the same exact stimuli. However, it is unclear whether the networks involved in the urge to blink are the same as those involved in the urge to perform a compulsion. Indeed, prior work comparing the urge to blink in controls and the urge to engage in mental compulsions in OCD identified some similarities but noted different time courses for the two types of urges (Brandt et al., 2018). The current study represents a first step toward investigating the neural basis of urges in OCD, and future imaging work should aim to compare urges related to OCD symptoms to other types of UFA.

Finally, the study had an uneven sample size with twice as many OCD patients as healthy controls, which could potentially lead to unequal variances between the groups. However, for all comparisons, we tested for unequal variance and used adjusted degrees of freedom when necessary, and none of these adjustments changed the significance of the results. Furthermore, post hoc testing between unmedicated OCD patients and controls—two groups with very similar sample sizes—revealed the same effects as when using the full sample of OCD patients. Thus, although the study's power would have been improved with a sample of controls as large as the sample of patients, the uneven sample sizes do not appear to have had a major impact on the reported effects.

In conclusion, despite the limitations, we found that OCD patients showed hyperactivity in several brain regions during early eyeblink suppression, including areas putatively related to the sensory experience of the urge itself (insula and mid cingulate) as well as those involved in visuospatial attention. Patients also made more erroneous blinks than controls during the suppression period, yet there were no areas of hypoactivation in OCD that could reflect a failure of inhibitory control systems. Future work should seek to further elucidate the neurobiology of urges in OCD as a critical step on route to developing interventions targeting these symptoms.

## CONFLICT OF INTEREST

The authors declare no potential conflict of interest.

## Supporting information


**Supplemental Table S1** P‐values for ROIs showing significant differences between OCD patients and controls (Cont) when a) covarying for depression (QIDS score), b) covarying for anxiety (BAI score), c) comparing 22 unmedicated OCD patients with 23 controls, and d) comparing 15 OCD patients without any comorbidities (“Only OCD”) with 23 controls. P‐values are corrected for false discovery rate across multiple ROI comparisons. All ROIs showed significant effects except for Cerebellum 4_5 (L) and Lateral Occipital Cortex, Superior (R), shown with asterisks, which were trend‐level significant (*p* < 0.10) for two comparisons.

**Supplemental Table S2** P‐values for ROIs showing significant differences between OCD patients and controls (Cont) when a) covarying for depression (QIDS score), b) covarying for anxiety (BAI score), c) comparing 22 unmedicated OCD patients 23 healthy controls, and d) comparing 15 OCD patients without any comorbidities (“Only OCD”) with 23 controls. P‐values are corrected for false discovery rate across multiple ROI comparisons. All ROIs showed significant effects except for Superior Parietal Lobule (R) and Lateral Occipital Cortex, Superior (L), shown with asterisks, which were trend‐level significant (*p* < 0.10) for two comparisons.

**Supplemental Table S3** Positive correlations with sensory phenomena scale (SPS) score in OCD patients.

**Supplemental Figure S1** Distribution of mean urge ratings for OCD patients (left, red bars) and controls (right, blue bars). OCD patients' ratings distribution was negatively skewed, with a greater proportion of patients giving higher urge ratings following blink suppression than controls.

## Data Availability

The data that support the findings of this study are available from the corresponding author upon reasonable request.
