# Supplementary material for: The buildup of an urge in obsessive–compulsive disorder: Behavioral and neuroimaging correlates
Source: Hum Brain Mapp. 2020 Jan 9;41(6):1611–25. doi: 10.1002/hbm.24898 (PMC7082184; doi:10.1002/hbm.24898)
Supplement: Supplementary file 1 — Supplemental Table S1 P‐values for ROIs showing significant differences between OCD patients and controls (Cont) when a) covarying for depression (QIDS score), b) covarying for anxiety (BAI score), c) comparing 22 unmedicated OCD patients with 23 controls, and d) comparing 15 OCD patients without any comorbidities (“Only OCD”) with 23 controls. P‐values are corrected for false discovery rate across multiple ROI comparisons. All ROIs showed significant effects except for Cerebellum 4_5 (L) and Lateral Occipital Cortex, Superior (R), shown with asterisks, which were trend‐level significant (p < 0.10) for two comparisons. [file HBM-41-1611-s001.docx]

**Supplemental Table 1.** P-values for ROIs showing significant differences between OCD patients and controls (Cont) when a) covarying for depression (QIDS score), b) covarying for anxiety (BAI score), c) comparing 22 unmedicated OCD patients with 23 controls, and d) comparing 15 OCD patients without any comorbidities (“Only OCD”) with 23 controls. P-values are corrected for false discovery rate across multiple ROI comparisons. All ROIs showed significant effects except for Cerebellum 4_5 (L) and Lateral Occipital Cortex, Superior (R), shown with asterisks, which were trend-level significant (p<0.10) for two comparisons.

| Harvard-Oxford Parcel | k | Covary QIDS | Covary BAI | Unmed OCD>  Cont | Only OCD>  Cont |
| --- | --- | --- | --- | --- | --- |
| ***Frontal/Insular*** |  |  |  |  |  |
| Frontal Orbital Cortex (L) | 20 | 0.0005 | 0.0149 | 0.0070 | 0.0355 |
| Insular Cortex (L) | 126 | 0.0001 | 0.0016 | 0.0007 | 0.0059 |
| Insular Cortex (L) | 42 | 0.0003 | 0.0019 | 0.0019 | 0.0169 |
| Cingulate Gyrus, Anterior (L) | 80 | 0.0006 | 0.0037 | 0.0015 | 0.0259 |
| ***Parietal*** |  |  |  |  |  |
| Postcentral Gyrus (R) | 28 | 0.0081 | 0.0074 | 0.0107 | 0.0158 |
| Angular Gyrus (L) | 245 | 0.0007 | 0.0016 | 0.0032 | 0.0122 |
| Angular Gyrus (R) | 315 | 0.0003 | 0.0014 | 0.0014 | 0.0059 |
| Superior Parietal Lobule (L) | 178 | 0.0006 | 0.0053 | 0.0015 | 0.0117 |
| Superior Parietal Lobule (R) | 218 | 0.0003 | 0.0067 | 0.0007 | 0.0093 |
| Supramarginal Gyrus, Anterior (L) | 128 | 0.0003 | 0.0026 | 0.0015 | 0.0071 |
| Supramarginal Gyrus, Posterior (L) | 323 | 0.0003 | 0.0014 | 0.0007 | 0.0052 |
| Supramarginal Gyrus, Posterior (R) | 464 | 0.0003 | 0.0016 | 0.0014 | 0.0058 |
| Lateral Occipital Cortex, Superior (L) | 620 | 0.0003 | 0.0014 | 0.0007 | 0.0078 |
| Lateral Occipital Cortex, Superior (R) | 337 | 0.0002 | 0.0016 | 0.0001 | 0.0059 |
| Cingulate Gyrus, Posterior (B) | 555 | 0.0003 | 0.0014 | 0.0007 | 0.0071 |
| Cingulate Gyrus, Posterior (B) | 28 | 0.0019 | 0.0070 | 0.0034 | 0.0036 |
| Precuneous Cortex (B) | 1702 | 0.0003 | 0.0014 | 0.0007 | 0.0036 |
| Precuneous Cortex (L) | 20 | 0.0056 | 0.0151 | 0.0063 | 0.0058 |
| ***Occipital*** |  |  |  |  |  |
| Intracalcarine Cortex (L) | 173 | 0.0033 | 0.0031 | 0.0027 | 0.0036 |
| Intracalcarine Cortex (R) | 72 | 0.0056 | 0.0074 | 0.0017 | 0.0048 |
| Intracalcarine Cortex (R) | 53 | 0.0013 | 0.0019 | 0.0017 | 0.0036 |
| Supracalcarine Cortex (R) | 41 | 0.0007 | 0.0016 | 0.0076 | 0.0059 |
| Supracalcarine Cortex (R) | 21 | 0.0019 | 0.0019 | 0.0017 | 0.0036 |
| Lingual Gyrus (L) | 120 | 0.0013 | 0.0026 | 0.0015 | 0.0036 |
| Lingual Gyrus (L) | 67 | 0.0013 | 0.0019 | 0.0046 | 0.0059 |
| Lingual Gyrus (R) | 119 | 0.0007 | 0.0017 | 0.0015 | 0.0052 |
| Lingual Gyrus (R) | 56 | 0.0006 | 0.0016 | 0.0015 | 0.0036 |
| Cuneal Cortex (L) | 198 | 0.0007 | 0.0025 | 0.0015 | 0.0058 |
| Cuneal Cortex (R) | 389 | 0.0006 | 0.0017 | 0.0019 | 0.0036 |
| Lateral Occipital Cortex, Inferior (L) | 316 | 0.0000 | 0.0014 | 0.0007 | 0.0052 |
| Lateral Occipital Cortex, Inferior (R) | 31 | 0.0005 | 0.0070 | 0.0010 | 0.0091 |
| Lateral Occipital Cortex, Superior (L) | 83 | 0.0003 | 0.0070 | 0.0017 | 0.0059 |
| Lateral Occipital Cortex, Superior (R) | 85 | 0.0009 | 0.0070 | 0.0020 | 0.0059 |
| Lateral Occipital Cortex, Superior (R) | 53 | 0.0027 | 0.0546* | 0.0017 | 0.0059 |
| Occipital Pole (L) | 265 | 0.0005 | 0.0016 | 0.0019 | 0.0036 |
| Occipital Pole (R) | 29 | 0.0020 | 0.0016 | 0.0113 | 0.0036 |
| ***Subcortical*** |  |  |  |  |  |
| Caudate (L) | 44 | 0.0003 | 0.0067 | 0.0019 | 0.0240 |
| Putamen (L) | 123 | 0.0002 | 0.0024 | 0.0007 | 0.0060 |
| Putamen (L) | 29 | 0.0002 | 0.0014 | 0.0021 | 0.0059 |
| Thalamus (L) | 31 | 0.0003 | 0.0014 | 0.0030 | 0.0203 |
| Hippocampus (L) | 73 | 0.0002 | 0.0014 | 0.0008 | 0.0059 |
| Cerebellum Crus 1 (R) | 181 | 0.0013 | 0.0053 | 0.0017 | 0.0206 |
| Cerebellum 4_5 (L) | 49 | 0.0044 | 0.0134 | 0.0266 | 0.0665* |
| Cerebellum 6 (L) | 217 | 0.0028 | 0.0073 | 0.0056 | 0.0322 |
| Cerebellum 6 (R) | 345 | 0.0007 | 0.0024 | 0.0017 | 0.0233 |
| Vermis 6 (B) | 51 | 0.0056 | 0.0079 | 0.0115 | 0.0338 |
| Vermis 7 (B) | 27 | 0.0065 | 0.0100 | 0.0114 | 0.0462 |

QIDS=Quick Inventory of Depressive Symptomatology; BAI=Beck Anxiety Inventory; Unmed=unmedicated; OCD=Obsessive-Compulsive Disorder; Cont=controls; k=cluster extent; R=right, L=left; B=bilateral. Clusters are listed in the same order as Table 2.
