# Supplementary material for: The buildup of an urge in obsessive–compulsive disorder: Behavioral and neuroimaging correlates
Source: Hum Brain Mapp. 2020 Jan 9;41(6):1611–25. doi: 10.1002/hbm.24898 (PMC7082184; doi:10.1002/hbm.24898)
Supplement: Supplementary file 3 — Supplemental Table S3 Positive correlations with sensory phenomena scale (SPS) score in OCD patients. [file HBM-41-1611-s003.docx]

**Supplemental Table 3.** Positive correlations with sensory phenomena scale (SPS) score in OCD patients.

| Label | BA | k | x | y | z | Max p |
| --- | --- | --- | --- | --- | --- | --- |
| Precentral/postcentral gyri (L) | 4, 6, 42, 43 | 36 | -60 | -8 | 10 | 0.0003 |
| Precentral/postcentral gyri (L) | 3, 4, 6 | 199 | -58 | -10 | 32 | 0.0005 |
| Supplementary motor area/paracentral lobule (R) | 4, 6 | 104 | 16 | -28 | 58 | 0.0005 |
| Insula (R) | 13, 41 | 34 | 36 | -22 | 12 | 0.0008 |
| Insula (R) | 13 | 20 | 46 | -10 | 12 | 0.0008 |
| Insula (L) | 13 | 39 | -36 | -14 | 18 | 0.0009 |
| Precentral/postcentral gyri (R) | 4, 42, 43 | 70 | 58 | -8 | 22 | 0.0011 |
| Precentral/postcentral gyri (R) | 6 | 31 | 56 | -8 | 36 | 0.0013 |

Labels are derived from the Automated Anatomical Labeling and Talairach Daemon databases as provided through xjview (v. 9.6, http://www.alivelearn.net/xjview). BA=Brodmann’s areas; k=cluster extent; Max p=maximum uncorrected p-value derived from permutation testing; R=right, L=left; coordinates are in MNI space. Clusters shown are within a mask consisting of insula, precentral and postcentral gyri, supplementary motor area, and paracentral lobule (see Brown et al., 2019).
