# Supplementary figures and images for: The buildup of an urge in obsessive–compulsive disorder: Behavioral and neuroimaging correlates
Source: Hum Brain Mapp. 2020 Jan 9;41(6):1611–25. doi: 10.1002/hbm.24898 (PMC7082184; doi:10.1002/hbm.24898)

## OCD

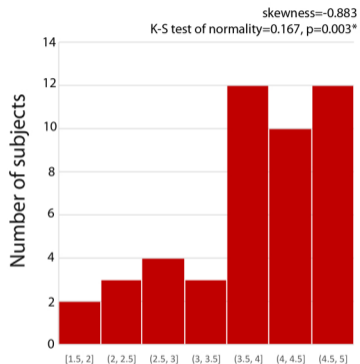

## Controls

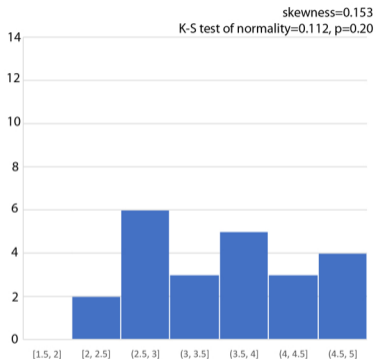

Urge ratings [bins]

Supplement: Supplementary file 4 — Supplemental Figure S1 Distribution of mean urge ratings for OCD patients (left, red bars) and controls (right, blue bars). OCD patients' ratings distribution was negatively skewed, with a greater proportion of patients giving higher urge ratings following blink suppression than controls. [file HBM-41-1611-s004.pdf]
